# Regulation of cellular quiescence by YAP/TAZ and Cyclin E1 in colon cancer cells: Implication in chemoresistance and cancer relapse

**DOI:** 10.18632/oncotarget.11057

**Published:** 2016-08-04

**Authors:** Matthieu Corvaisier, Marjolaine Bauzone, François Corfiotti, Florence Renaud, Mehdi El Amrani, Didier Monté, Stéphanie Truant, Emmanuelle Leteurtre, Pierre Formstecher, Isabelle Van Seuningen, Christian Gespach, Guillemette Huet

**Affiliations:** ^1^ University Lille, Inserm, CHU Lille, UMR-S1172-JPARC-Jean-Pierre Aubert Research Center, F-59000, Lille, France; ^2^ Department of Digestive Surgery and Transplantation, CHRU Lille, F-59000, Lille, France; ^3^ Center of Biology-Pathology, CHRU Lille, F-59000, Lille, France; ^4^ UMR8576 CNRS-Université de Lille Nord de France, F-59658, Villeneuve d'Ascq, France; ^5^ INSERM U938, “Molecular and Clinical Oncology”, Hôpital Saint-Antoine, University Pierre et Marie Curie, F-75012, Paris, France

**Keywords:** c-Myc, CREB, stemness, liver metastases, prognosis

## Abstract

Our aim was to decipher the role and clinical relevance of the YAP/TAZ transcriptional coactivators in the regulation of the proliferation/quiescence balance in human colon cancer cells (CCC) and survival after 5FU-based chemotherapy. The prognostic value of YAP/TAZ on tumor relapse and overall survival was assessed in a five-year follow-up study using specimens of liver metastases (*n* = 70) from colon cancer patients. In 5FU-chemoresistant HT29-5F31 and -chemosensitive HCT116 and RKO CCC, a reversible G0 quiescent state mediated by Cyclin E1 down-regulation was induced by 5FU in 5F31 cells and recapitulated in CCC by either YAP/TAZ or Cyclin E1 siRNAs or the YAP inhibitor Verteporfin. Conversely, the constitutive active YAPdc-S127A mutant restricted cellular quiescence in 5FU-treated 5F31 cells and sustained high Cyclin E1 levels through CREB Ser-133 phosphorylation and activation. In colon cancer patients, high YAP/TAZ level in residual liver metastases correlated with the proliferation marker Ki-67 (*p* < 0.0001), high level of the YAP target CTGF (*p* = 0.01), shorter disease-free and overall survival (*p* = 0.008 and 0.04, respectively). By multivariate analysis and Cox regression model, the YAP/TAZ level was an independent factor of overall (Hazard ratio [CI 95%] 2.06 (1.02–4.16) *p* = 0.045) and disease-free survival (Hazard ratio [CI 95%] 1.98 (1.01–3.86) *p* = 0.045). Thus, YAP/ TAZ pathways contribute to the proliferation/quiescence switch during 5FU treatment according to the concerted regulation of Cyclin E1 and CREB. These findings provide a rationale for therapeutic interventions targeting these transcriptional regulators in patients with residual chemoresistant liver metastases expressing high YAP/TAZ levels.

## INTRODUCTION

Cancer relapse after removal of the primary tumour and adjuvant therapy can occur after years or even decades of tumour remission. This latency is linked to the presence of residual disseminated tumour cells that have entered dormancy and escaped therapies. Our previous work in 5FU-chemoresistant HT29 human colon cancer cells and clinical liver metastases has raised the issue of the Yes-associated protein YAP as a potential regulator in the dormancy/growth transition during 5FU-based chemotherapy [1].

In the Hippo pathways, YAP and its paralog TAZ (transcriptional co-activator with PDZ-binding motif) are two critical oncogenic components acting through their interactions with transcriptional enhancer associate domain (TEAD1-4) family transcription factors and DNA binding partners to promote target genes involved in cancer progression [2–5]. YAP and TAZ transcriptional activity is inhibited by the large tumor suppressor kinases (LATS1/2) that are phosphorylated and activated by the MST1/2 kinases, and a vast array of upstream endogenous and exogenous Hippo pathway regulators, including metabolic and nutrient signals, membrane receptors, E-cadherin -dependent cell-cell adhesion and hypoxia [6]. Phosphorylation of YAP at Ser-127 by LATS1/2 results in 14-3-3 binding, YAP cytoplasmic retention or degradation via YAP ubiquitination by the SCF (β-TRCP) E3 ubiquitin ligase [7]. Thus, this Hippo pathway kinases cascade and upstream activators are considered as tumor suppressors acting through inhibitory signals on the YAP/TAZ transcriptional co-activators [8–11]. Another level of complexity is reported with signaling crosstalk between Hippo and the cancer-related pathways Wnt, TGFβ/SMAD and DNA damage responses [6]. Accordingly, YAP and TAZ are described to integrate tumor growth, stem cell-like cancer cell renewal, invasive growth and metastasis in various tumors, including colorectal cancers [12–16]. In addition, several reports also indicate that YAP and TAZ modulate mesenchymal stem cell differentiation and epithelial-mesenchymal transition (EMT) linked to invasive growth, stem cell like phenotypes and chemoresistance [17–22]. Consistently, recent studies support a role of YAP/TAZ signaling in invasion [4] and chemoresistance to RAF and MEK inhibitors and other anticancer agents [23–25].

Although YAP and TAZ signaling emerges now as a critical determinant in tumorigenesis, our knowledge of the mechanisms underlying the implication of these transcriptional cofactors in cancer cell chemoresistance, quiescence and survival is still limited. Our previous study in 5FU-chemoresistant human colon cancer cells supported the notion that 5FU-induced cellular quiescence is associated with the concomitant depletion of nuclear YAP levels, but provided no mechanistic link. This data prompted us to examine whether YAP regulates cellular quiescence connected to 5FU-mediated chemoresistance, stemness and survival of HT29-derived 5F31 human colon cancer cells. In 5FU-chemoresistant 5F31 cells and chemosensitive HCT116 and RKO cells, we report that the G0 pool of resting cells is increased by dual YAP/TAZ silencing. Conversely, ectopic expression of a dominant constitutive active mutant Ser127Ala YAPdc in 5F31 cells restricts 5FU-induced cellular quiescence through the oncogenic activation of the cAMP response element binding protein (CREB). According to these experimental data, a complementary clinical study on invasive growth and tumor relapse was undertaken in liver metastases from colon cancer patients according to the molecular markers YAP/TAZ, Ki-67 and the YAP target genes *CTGF*, *AXL* and *Cyr61*. Taken together, our data identify a key role for YAP/TAZ connected with Cyclin E1/ c-Myc and CREB signaling cascades in the regulation of 5FU chemoresistance at the proliferation/quiescence switch in colon cancer cells, according to their impact on tumor dormancy/recurrence, disease-free and overall survival in patients with residual liver metastases.

## RESULTS

### Differential regulation of cellular quiescence by YAP inhibition and activation in human colon cancer cells

**Figure 1:**
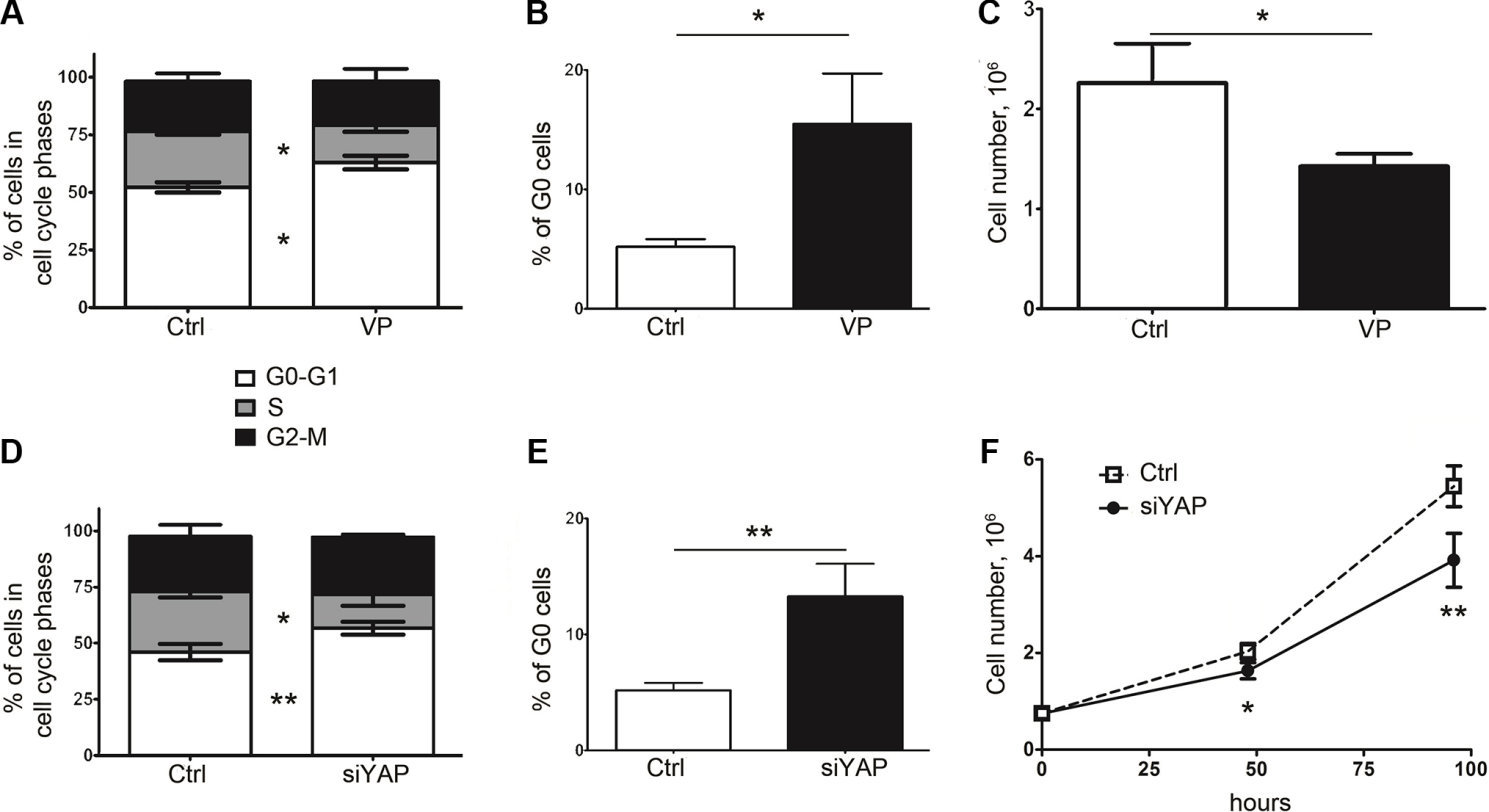
Inhibition of YAP expression or activity in 5F31 is associated with cellular quiescence (**A**, **B**) Analysis of cell cycle distribution (G0-G1, S and G2-M phases) and percentage of G0 resting cells in 5F31 cells incubated in the presence and absence (control: Ctrl) of the YAP inhibitor Verteporfin (VP). Cells were treated by 10 μM VP for 48 hours and processed by flow cytometry for G0-G1, S, G2-M distribution and quantification of G0 phase cells using Ki-67 labelling. (**C**) Cell count after 48 hour treatment by 10 μM VP. (**D**, **E**) Flow cytometry analysis of cell cycle distribution (G0-G1, S and G2-M phases) and percentage of G0 quiescent cells in YAP-silenced *vs* control 5F31 cells. Cells were treated for 48 hours by 30 nM YAP siRNA or nontargeting siRNA (Ctrl cells). (**F**) Cell growth of YAP-silenced *vs* control 5F31 cells after 48 hour treatment by siRNA. All data are from 3 replicates.

In order to establish a mechanistic link between entry into cellular quiescence and the YAP status in human colon cancer cells, we first examined the impact of YAP inhibition on cell cycle progression and clonogenic survival. Verteporfin (VP), initially used in the photodynamic therapy of age-related macular degeneration, recently emerged as an inhibitor of YAP/ TEAD interaction and YAP/TEAD transcriptional activity [26]. When 5FU-chemoresistant 5F31 cells were exposed to 10 μM VP, we observed an alteration in the cell cycle pattern marked by a decrease in the S phase and an increase in G0–G1 phase, as shown in Figure 1A (*p* < 0.05). Flow cytometry analysis of cellular quiescence using exclusion of Ki-67 labelling in G0 cells showed that VP increased the pool of G0 quiescent cells from 4.9 ± 0.9% in control cells (Ctrl) to 15.8 ± 2.9% in VP-treated cells, *p* < 0.05 (Figure 1B). In agreement, cell growth was decreased by 35.5 ± 14.1% after 48 hours of VP treatment (Figure 1C). Interestingly, YAP knockdown using YAP siRNA also increased the G0 pool (5.2 ± 0.6% in control cells versu*s* 13.3 ± 2.8% in siYAP cells, *p* < 0.01) and decreased the number of cells in the S-phase and cell growth without change in cell viability and SubG1 cells (Figure 1D–1F and data not shown). Of note, YAP knockdown led to a decrease in the size and number (by 2-fold) of spheres and cancer stem cell markers ALDH1A3, CD133 and Lgr5, with no change in CD44 (Supplementary Figure S1).

**Figure 2:**
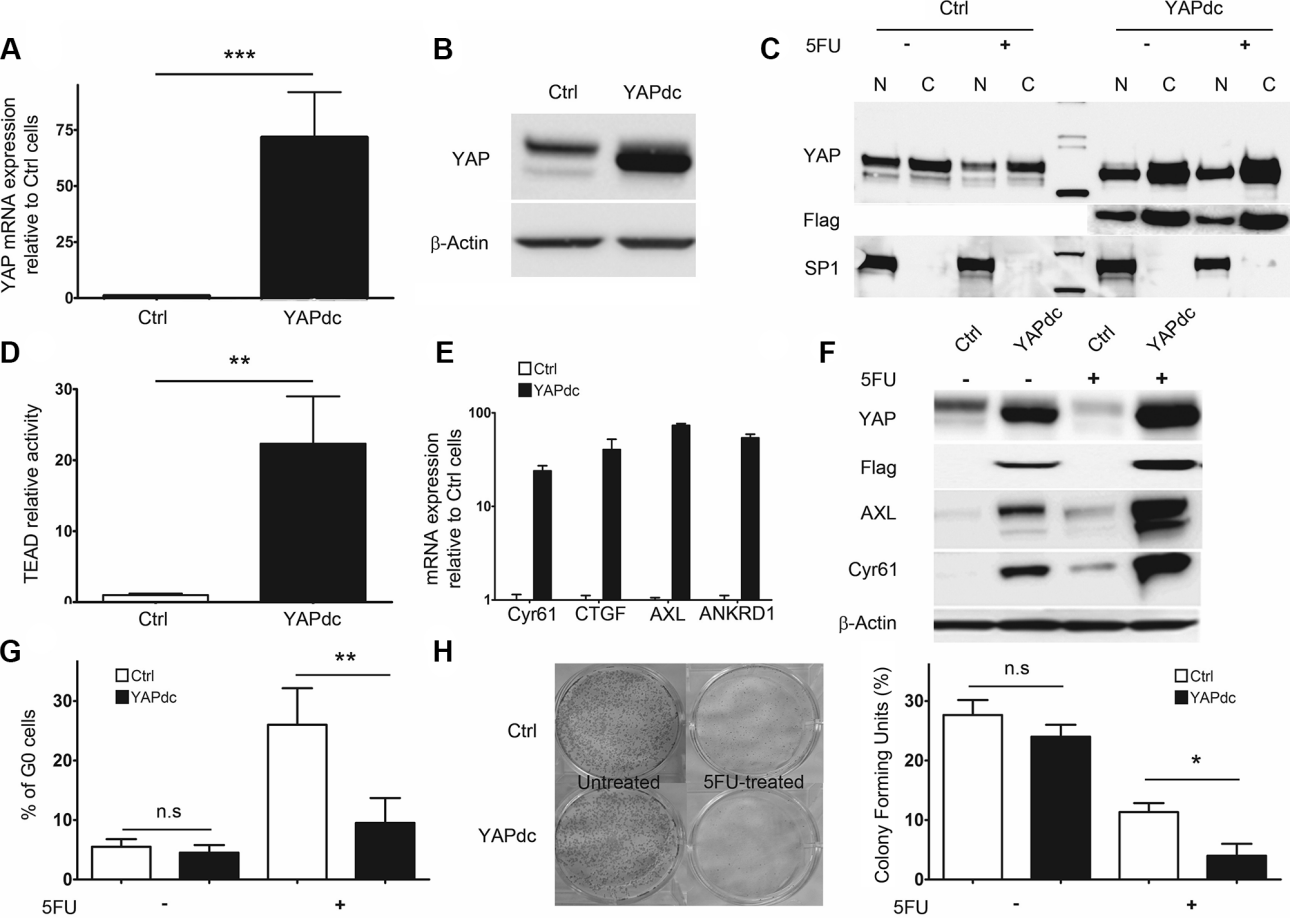
Constitutively active nuclear YAPdc restricts cellular quiescence in 5F31 (**A**, **B**) Ectopic expression of YAPdc by RT-qPCR and Western blot in control vector (Ctrl)- and YAPdc-transfected 5F31 cells. (**C**) Immunoblots using antibodies directed against YAP, the Flag epitope and Sp1 in nuclear (N) and cytoplasmic (C) fractions from untreated and 5FU-treated (40 μM, 96 hours) YAPdc and 5F31 cells. (**D**) transcriptional activity of the TEAD-luciferase reporter gene in control and YAPdc 5F31 cells. (**E** and **F**) Expression of the YAP target genes *Cyr61*, *CTGF*, *AXL* and *ANKRD1* by RT-qPCR and Western blotting in control and YAPdc 5F31 cells. (**G**) Percentage of cells in the G0 phase after 96 hour 5FU-treatment (40 μM) in control and YAPdc 5F31 cells. Control vector and YAPdc-transfected cells were incubated for 96 hours in the presence or absence of 5FU and processed by flow cytometry analysis for quantification of G0 phase cells using Ki-67 labelling. Data are from 5 replicates. (**H**) Colony forming units assay of untreated and 5FU-treated YAPdc and control 5F31 cells. Western blots are representative of 3 experiments.

In order to gain further insight into the role of YAP in the proliferation/quiescence balance, we generated 5F31 cells stably transfected with a dominant constitutive nuclear YAPdc (Flag-YAP S127A). The mutation of the 127-Serine residue prevents YAP phosphorylation by the Hippo pathway and promotes its nuclear accumulation. As expected, high YAP transcript and protein levels were detected in YAPdc-transfected 5F31 cells (Figure 2A–2B). Isolation of nuclear and cytosolic fractions showed that high level of ectopic Flag-YAP was targeted in the nucleus (Figure 2C). In 5FU-treated 5F31 cells, endogenous nuclear YAP protein markedly decreased whereas in 5FU-treated YAPdc 5F31 cells, ectopic Flag-YAPdc was maintained at high level in the nuclei. As expected, a marked increase (by 23-fold, *p* < 0.01) in TEAD transcriptional activity was measured in YAPdc cells (Figure 2D). In agreement, the YAP target genes *CYR61, CTGF*, *AXL* and *ANKRD1* were strongly upregulated in YAPdc-transfected 5F31 cells (Figure 2E). Consistently, both AXL and Cyr61 proteins were upregulated at high levels by 5FU in YAPdc cells, and lower levels in 5F31 cells (Figure 2F) suggesting YAP-independent upregulation of AXL and Cyr61 by 5FU. Most interestingly, cellular quiescence was reduced by more than 2-fold in 5FU-treated YAPdc cells (9.5 ± 4.2% G0 cells) as compared to 5FU-treated 5F31 cells (26.1 ± 6.2%, *p* < 0.01, Figure 2G). Thus, our data indicate that in 5FU-treated 5F31 cells, YAPdc is a limiting factor for the entry or exit of 5F31 cells at the reversible G0 quiescent state (RQS). Currently, cellular quiescence accounts for a possible mechanism of chemoresistance as the activity of cytotoxic agents is greatly reduced in quiescent cells not engaged in the cell cycle [27–29]. In order to examine the impact of quiescence on chemoresistance, we next compared YAPdc and control 5F31 cells after 96 hour exposure to 5FU, using the clonogenic cell survival assay (Figure 2H). Interestingly, the ratio of colonies formed after 5FU exposure was of 11.3 ± 1.5% in control 5F31 cells *vs* 4.2 ± 2.1% in YAPdc cells (*p* < 0.05). Thus, the limitation of the quiescent state induced by ectopic YAPdc is associated to a decreased cell survival response to 5FU-exposure (Figure 2H). Our data reveal that YAP plays a critical role in the quiescence/proliferation balance in 5FU-chemoresistant 5F31 cells.

### Induced G0 state in 5FU-treated 5F31 cells is characterized by decreased Cyclin E1 and c-Myc levels

**Figure 3:**
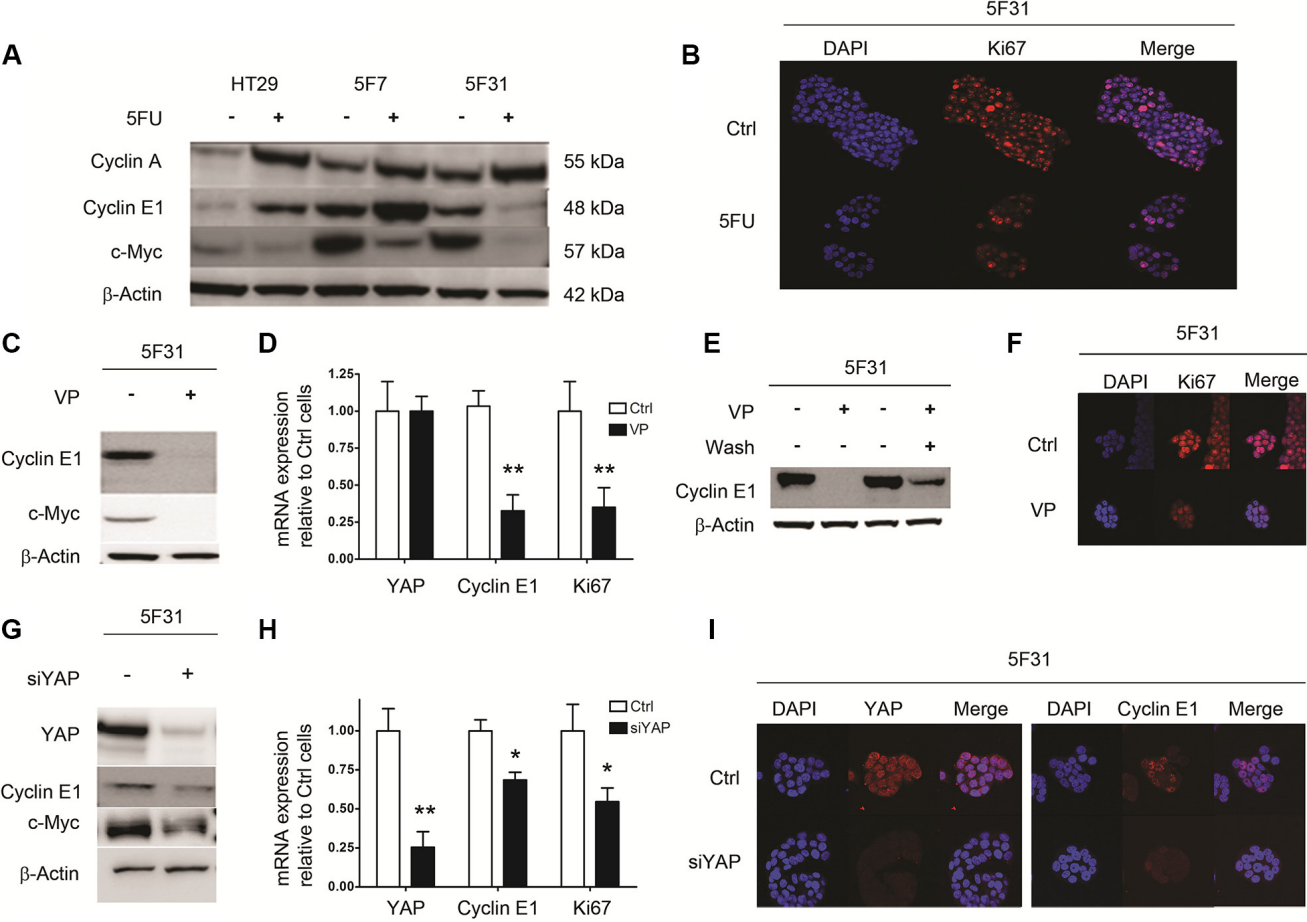
Cellular quiescence induced by 5FU, VP and YAP knockdown in 5F31 cells is associated to decreased in Cyclin E1 levels (**A**) Western blot analysis of Cyclins A1, E1 and c-Myc after 96 hours 5FU treatment of HT29, 5F7 and 5F31 cells. (**B**) Confocal microscopy analysis of Ki-67 expression in control and 5FU-treated (40 μM) 5F31 cells (magnification ×40). (**C**, **D**) Cyclin E1, c-Myc and Ki-67 transcript and protein levels after 48 hours VP treatment (10 μM) of 5F31 cells. (**E**) Reversibility of Cyclin E1 down-regulation after VP treatment. (**F**) Confocal microscopy analysis of Ki-67 expression in control and VP-treated 5F31 cells (magnification ×40). (**G**, **H**) Cyclin E1, c-Myc and Ki-67 transcript and protein levels after YAP-silencing by 30 nM YAP siRNA in 5F31 cells. Control cells are transfected with non-targeting sequence. (**I**) Confocal microscopy analysis of Cyclin E1 in control and YAP-silenced 5F31 cells by 30 nM YAP siRNA (magnification ×63). All experiments were reproduced at least three times.

In order to understand the mechanisms responsible for the regulation of the G0 resting phase by 5FU, we analyzed the status of the cell cycle regulators c-Myc, Cyclin A and E1. Under 5FU-exposure, we compared 5F31 cells with HT29 and 5F7 cells that are not induced in G0 phase but accumulate in the S and G2-M phases of the cell cycle [1]. As shown in Figures 3A, 5FU-treatment increased Cyclin A and decreased c-Myc levels in all cell types. Interestingly, 5FU selectively down-regulated Cyclin E1 in 5FU-treated 5F31 cells. However, the same treatment increased Cyclin E1 in HT29 and 5F7 cells. Accordingly, nuclear exclusion of Ki-67 protein was observed in 5FU-treated 5F31 cells (Figure 3B). Then, we examined the status of Cyclin E1, c-Myc and Ki-67 in 5F31 cells induced into cellular quiescence by VP or YAP-siRNA treatment. The YAP inhibitor VP induced a remarkable loss of Cyclin E1 and c-Myc proteins, both associated with down-regulation of Cyclin E1 and Ki-67 transcripts. In contrast, YAP-mRNA levels were not affected by this YAP inhibitor (Figure 3C–3D). As observed for the reversible quiescent state (RQS) induced by 5FU in 5F31 cells [1], depletion of Cyclin E1 protein by VP was reversible (Figure 3E). Our immunofluorescence experiments indicate that the Ki-67 nuclear antigen was undetectable following VP and YAP-siRNA treatment (Figure 3F). Similarly, YAP knockdown produced a marked decrease in Cyclin E1 (38 ± 3%) and c-Myc levels (40 ± 5%) and decreased the transcripts encoding YAP, Cyclin E1 and Ki-67 (Figure 3G–3H). As expected, YAP silencing depleted the Cyr61, AXL and CTGF transcripts (data not shown). Confocal microscopy analysis of YAP-silenced 5F31 cells showed that Cyclin E1 was localized in both the nucleus and cytoplasm in control 5F31 cells whereas it was only found in the cytoplasm of YAP-silenced 5F31 cells (Figure 3I). Thus, YAP regulates the two cell cycle regulators Cyclin E1 and c-Myc.

### YAP/TAZ and Cyclin E1 knockdown induce cellular quiescence in HCT116 and RKO human colon cancer cells

**Figure 4:**
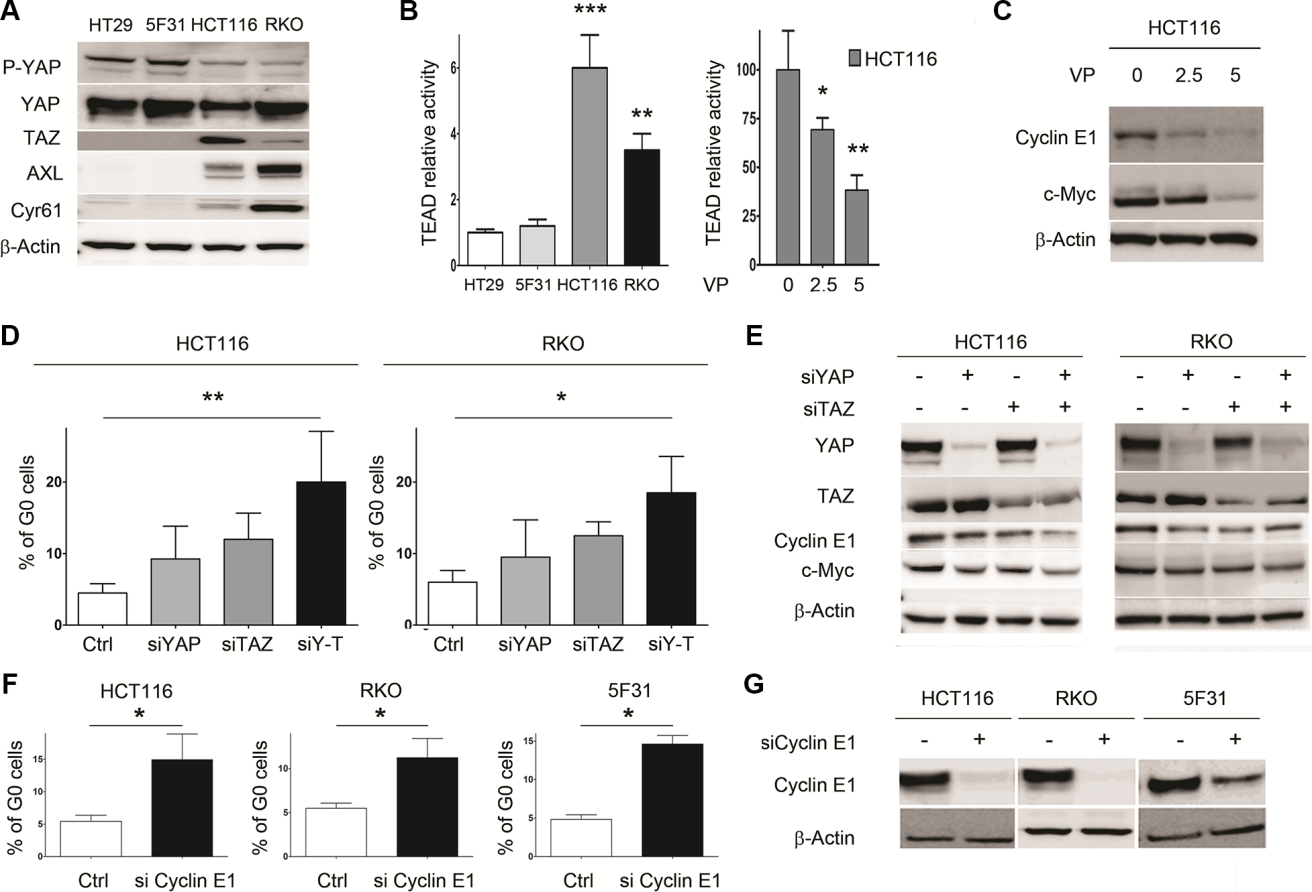
Dual knockdown of the YAP/TAZ co-activators in HCT116 and RKO cells is associated with cellular quiescence and decreased Cyclin E1 levels (**A**) Expression of YAP, P-YAP (Ser127), TAZ, Cyr61 and AXL analyzed by Western blotting. B- TEAD relative activity in control cells (HT29, 5F31, HCT116, RKO) and VP-treated HCT116 cells (2.5 and 5 μM VP). (**B**, **C**) Impact of VP treatment on TEAD activity and Cyclin E1 and c-Myc levels in HCT116 cells. (**D**) Percentage of G0 cells in YAP-, TAZ- and YAP/TAZ-silenced HCT116 and RKO cells. Cells were treated by 15 nM YAP siRNA and/or 15 nM TAZ siRNA and processed for the quantification of G0 phase cells by flow cytometry using Ki-67 labelling. Control cells are transfected with non-targeting sequence. Data are from 3 replicates. (**E**) Effect of YAP-, TAZ- and YAP/TAZ-silencing on Cyclin E1 and c-Myc levels in HCT116 and RKO cells. (**F**, **G**) Effect of Cyclin E1 silencing on cellular quiescence in 5F31, HCT116 and RKO cells. Cyclin E1 siRNA concentration was 30 nM for 5F31 cells and 15 nM for HCT116 and RKO cells. Control cells are transfected with nontargeting sequence. Control and silenced cells were then processed for the quantification of G0 phase cells by flow cytometry using Ki-67 labelling. Data are from 3 replicates. Western blots are representative of at least 3 experiments.

Next, we examined the role of YAP/TAZ on cellular quiescence in 5FU-sensitive RKO and HCT116 cells. As shown in Figure 4A, endogenous YAP was expressed at high levels in HT29, RKO cells and at lower level in HCT116 cells. Interestingly, TAZ-proficient HCT116 and RKO cells expressed low levels of YAP phosphorylation at position 127 as compared to HT29 and 5F31 cells. In contrast, both HT29 cells and its clonal derivative 5F31 are TAZ-deficient. Accordingly, higher TEAD transcriptional activity and expression of the YAP targets AXL/Cyr61 were observed in HCT116 and RKO cells (Figure 4A–4B). In HCT116 cells, the YAP inhibitor VP inhibited TEAD transcriptional activity in a concentration-dependant manner (2.5–5 μM) and strongly decreased c-Myc and Cyclin E1 levels (Figure 4B–4C). Dual YAP/TAZ knockdown increased the proportion of G0 cells from 4.44 ± 1.1% to 20.5 ± 7.2% in HCT116 cells, and from 6.1 ± 1.8% to 18.9 ± 5.1% in RKO cells (Figure 4D). Similar accumulation of G0 cells was observed in HCT116 cells treated with the YAP inhibitor VP (data not shown). Consistently, YAP/TAZ dual silencing reduced both Cyclin E1 (by 51 ± 8% and 32 ± 3% in HCT116 and RKO cells, respectively) and c-Myc protein levels (by 49 ± 7% and 33 ± 5% in HCT116 and RKO cells, respectively) (Figure 4E). Our data demonstrate that inhibiting YAP/TAZ expression or activity in colon cancer cells promotes the resting G0 quiescent state connected with Cyclin E1 and c-Myc down-regulation.

Next, we analysed the impact of Cyclin E1 depletion on cellular quiescence in 5F31, HCT116 and RKO cells (Figure 4F–4G). Interestingly, Cyclin E1 siRNA increased the percentage of G0 quiescence in HCT116 (from 5.3 ± 0.7 to 14.3 ± 3.5%), RKO (from 5.8 ± 0.8 to 12.3 ± 1.7%) and 5F31 cells (4.7± 0.5 to 13.5 ± 1.2%) as shown in Figure 4F–4G. Thus, Cyclin E1 plays a critical role in the induction of the G0 status promoted by 5FU, YAP/TAZ knockdown and YAP inhibition by VP.

### YAPdc counteracts 5FU-induced quiescence and Cyclin E1 down-regulation through CREB activation

**Figure 5:**
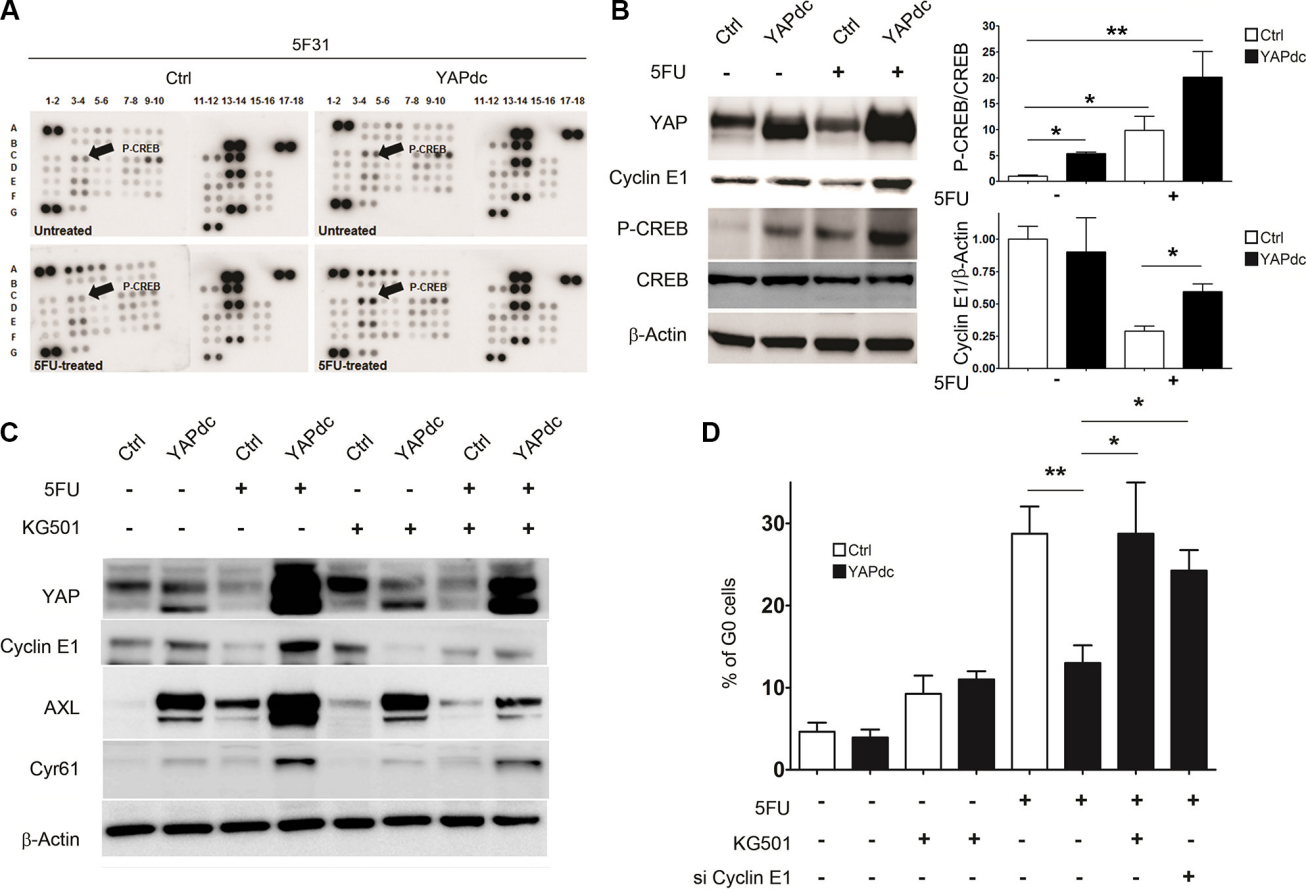
Ectopic expression of YAPdc alleviates cellular quiescence induced by 5FU (**A**) Total cell lysates from untreated and 5FU-treated (96 hours, 40 μM) control (Ctrl) and YAPdc 5F31 cells were analyzed by phospho-kinase arrays. Cyclic AMP -Response Element-Binding protein (CREB), c-Yes tyrosine kinase (arrows, see the phospho-array coordinates in Supplementary Materials and Methods). F17, F18, G5, G6: negative controls; A1, A2, A17, A18, G1, G2: positive controls. (**B**) Western blot analysis of YAP, Cyclin E1, P(Ser133)-CREB and CREB. Actin was used as loading control. Data are representative of 3 experiments. (**C**) Effect of the CREB inhibitor KG-501 on Cyclin E1, Cyr61 and AXL levels in control vector (Ctrl)- and YAPdc-tranfected 5F31 cells incubated in the presence or absence of 5FU (40 μM, 96 hours). KG-501 was used at the concentration of 20 μM for the last 48 hours of the experiment. Data are representative of 3 experiments. (**D**) Effect of KG-501 on cellular quiescence in control and 5FU-treated 5F31 and YAPdc cells, and impact of Cyclin E1 knockdown on cellular quiescence in 5FU-treated YAPdc 5F31 cells. Data are from 3 replicates.

Since nuclear YAP activation restricts cellular quiescence induced by 5FU in 5F31 cells (Figure 2G), we analyzed the signaling pathways regulated by 5FU and YAPdc (Figure 5A). Of note, the phosphokinase array demonstrates that both YAPdc and 5FU induced Ser133 CREB phosphorylation (Figure 5A). Western blotting confirmed that CREB phosphorylation was increased 5- and 9-fold by YAPdc and 5FU in 5F31 cells, respectively (Figure 5B). Most importantly, combination treatment revealed the reciprocal interaction between YAPdc and 5FU on CREB activation since the P-CREB/CREB ratio increased 20-fold, versus control cells (Figure 5B). Most importantly, YAPdc counteracted the depletion of Cyclin E1 observed during cellular quiescence induced by 5FU. In order to delineate the role of CREB in the reversion of cellular quiescence induced by 5FU in 5F31 YAPdc cells, we analyzed the impact of the KG-501 inhibitor that disrupts the CREB/CBP complex. We observed that KG-501 prevented the increased expression of Cyclin E1, AXL and Cyr61 in 5F31 YAPdc cells treated by 5FU (Figure 5C). Accordingly, both Cyclin E1 silencing and KG-501 restored the quiescence response in 5FU-treated YAPdc cells (Figure 5D). These results show that CREB signaling maintains YAPdc- transfected 5F31 cells in a cycling state under 5FU-treatment.

### High YAP/TAZ levels in residual metastases from colon cancer patients correlate with Ki-67, shorter disease-free survival and overall survival

**Figure 6:**
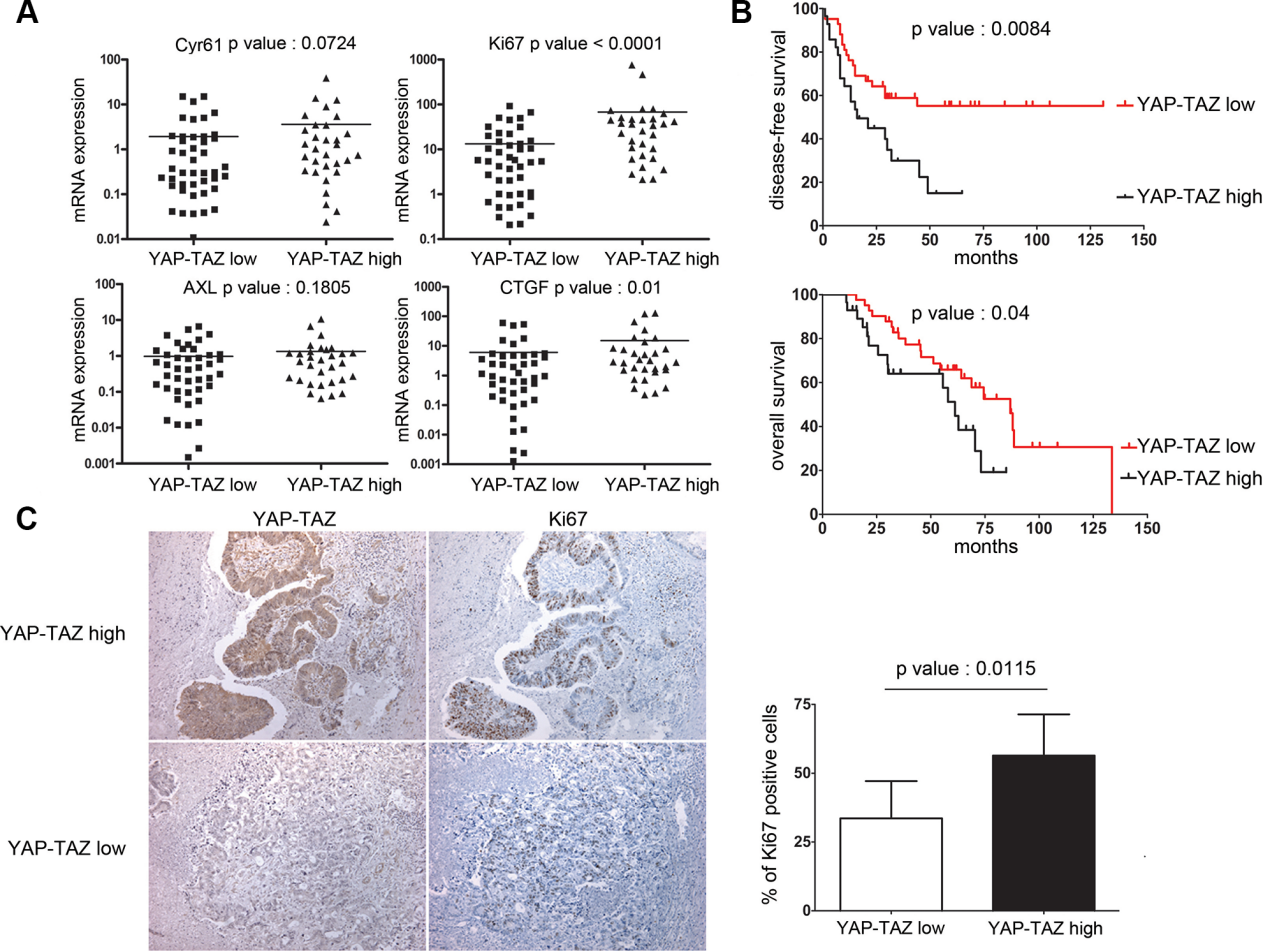
YAP/TAZ levels in liver metastases are highly correlated with Ki-67 and tumor relapse in colon cancer patients (**A**) Comparisons between the transcript levels of Ki-67, CTGF, Cyr61 and the AXL median transcript levels in the two liver metastases subgroups, *ie* YAP/TAZ low and YAP/TAZ high (description in Supplementary Material and Methods). (**B**) Kaplan-Meier survival analysis of colon cancer patients with liver metastases, according to the combined expression of YAP/TAZ transcripts. (**C**) Ki-67 and YAP/TAZ immunohistochemistry in liver metastases and analysis of the correlation between the percentages of Ki67 immunoreactive cells and nuclear YAP/TAZ staining (magnification × 100).

To examine the relevance of YAP and TAZ in the control of cellular quiescence and growth of disseminated colon tumor cells, we have compared expression of YAP/ TAZ and Ki-67, Cyr61, AXL, and CTGF using RT-qPCR in liver metastases resected from 70 colon cancer patients (Supplementary Table S1). Two groups of patients were discriminated according to the levels of YAP and TAZ transcripts, *i.e*. “YAP-TAZ Low” and “YAP-TAZ High” levels. Our data revealed a strong correlation between these two groups according to the Ki-67 marker, with high Ki-67 levels in YAP-TAZ High group and low Ki-67 levels in YAP-TAZ Low group (*p* < 0.0001, Figure 6A). YAP levels correlated with CTGF (*p* = 0.01), showed a trend with Cyr61 (*p* = 0.0724), but were not found to correlate with AXL (*p* = 0.1805). Our data suggests a decisive role of YAP/TAZ in the control of the proliferation/quiescence switch in liver metastases.

Most importantly, YAP-TAZ negatively correlated with the disease-free survival with high significance (*p* = 0.008, Figure 6B, and Supplementary Table S2). By multivariate survival analysis, YAP-TAZ significantly correlated with a shorter post-surgical disease-free survival, independently of the neoadjuvant chemotherapy and synchronous metastatic disease, with a higher risk for relapse (Hazard Ratio [CI95%] 1.98 (1.01–3.86) Supplementary Table S3). Moreover, YAP-TAZ also correlates negatively with the overall survival of patients (*p* = 0.04, Figure 6B, and Supplementary Table S4) with a higher risk of death (Cox regression, Hazard Ratio [CI95%] 2.06 (1.02–4.16) Supplementary Table S5). Examination of liver metastases by immunohistochemistry also showed that nuclear YAP-TAZ correlated with a higher percentage of Ki-67 -positive cancer cells (55% versus 32%, *p* = 0.0115 Figure 6C). Consistent with our experimental studies showing that YAP/TAZ sustains cycling of colon cancer cells, our clinical data show that these two co-activators play a crucial role in the deregulated growth controls inherent to disseminated human colon cancer cells in liver metastases.

## DISCUSSION

Current therapeutic strategies directed against epithelial human solid tumors are mostly based on classical cytotoxic and genotoxic drugs, radiotherapeutic interventions and newly designed anticancer agents targeting signaling and metabolic pathways. These therapeutic strategies relate on their ability to disrupt cancer cell survival, invasive growth, tumor angiogenesis and metastasis. Accordingly, anticancer drug resistances are governed by multifactorial molecular and cellular mechanisms induced in both cancer cells and the stromal compartments of growing epithelial tumors [30]. In the present study, our results identify a new mechanism by which YAP regulates 5FU-chemoresistance via the accumulation of human colon cancer cells HT29 (5F31) into the G0 quiescent phase of the cell cycle. This reversible cellular quiescence induced by 5FU is mediated by nuclear YAP depletion and selective down-regulation of Cyclin E1. In 5FU-chemosensitive HCT116 and RKO cells expressing both YAP and TAZ at high levels, cellular quiescence was recapitulated by YAP/TAZ co-silencing. In these two models, Cyclin E1 knockdown was linked to increased G0 phase cells, supporting a more spread role for YAP and the cell cycle regulator Cyclin E1 in chemoresistance and cellular quiescence.

While the Hippo/YAP pathways were initially thought to control organ size and growth, recent reports support their implication in the regulation of cancer cell proliferation via the transcriptional activation of many cell proliferation-related genes and direct regulation of the cell cycle machinery [3]. An additional level of complexity is illustrated by molecular crosstalk between YAP/TAZ-TEAD and JUN/FOS –AP-1 DNA binding sites acting at cis-regulatory distal enhancers of target genes involved in cell cycle control and tumor growth [31]. These cell cycle and proliferation genes encode transcriptional regulators (Ets1, c-Myc and Mybl1), Cyclins and their activators, and other factors required for completion of mitosis. In breast cancer cells, recent data demonstrate that YAP depletion by miR-506 disrupts the cell cycle through a G0/G1 phase arrest and reduction of S-phase and G2-M phase cells [32]. Consistently, Cyclin E1 down-regulation induced by 5FU in 5F31 cells is a critical quiescence signal, downstream of nuclear YAP depletion. Redundant and divergent functions of Cyclins E1/E2 are described during development and cancer [33]. The canonical function of Cyclins E1/E2 is to promote S-phase entry through Cyclin E/cdk2 phosphorylation of the retinoblastoma protein pRb1 that releases E2F transcriptional activity [34]. The generation of Cyclin E1- and E2- deficient mice identified a redundant kinase-independent function of Cyclins E1 and E2 during development [35–37]. Interestingly, Cyclin E1 facilitates a non-redundant and rapid cell cycle re-entry from the quiescent G0 state of hematopoietic stem cells following myeloblastive stress induced by 5FU [38]. In our study, down-regulation of Cyclin E1 levels resulting from exposition to 5FU or YAP/TAZ depletion brings cells into a reversible G0 quiescent state. Of note, c-Myc was down-regulated during induced quiescence in both 5F31 cells and 5FU-chemosensitive HCT116 and RKO cells. c-Myc is an important YAP/TAZ effector in cell proliferation, which however could not recapitulate *per se* the effects of YAP/TAZ [31]. It is known that *CCNE1* and *CCNE2* genes are directly or indirectly controlled by c-Myc pathways during mouse development and breast tumors driven by c-*Myc* [39].

Our data support the notion that 5FU-induced chemoresistance and quiescence in human colon cancer cells are regulated by a fine tuning of YAP abundance and nuclear activity connected with the concomitant down-regulation of Cyclin E1 and c-Myc. Accordingly, both YAP and Cyclin E1, but not YAPdc, are subjected to proteasome degradation in our experiments related to 5FU-mediated cellular quiescence (Figure 5B and Supplementary Figure S2). In the presence of the proteasome inhibitor MG132, YAP and Cyclin E1 levels were kept at their initial levels in 5FU-treated 5F31 cells, highlighting a potential role of the proteasome in the regulation of cellular quiescence. In a mouse model of chronic myeloid leukemia, c-Myc downregulation by the Fbw7 ubiquitin ligase plays a pivotal role towards quiescence in Leukemia Initiating Cells (LICs) resistant to Imatinib [40]. Accordingly, *Fbw7* genetic ablation induced cell cycle re-entry in quiescent LICs and restored Imatinib sensitivity. In this context, our data support the notion that 5FU-resistant colon cancer cells enter into quiescence following 5FU treatment and subsequent proteasomal degradation of YAP and Cyclin E as direct substrates of the β-TrCP and Fbw7 ubiquitin ligases, respectively [7, 41]. Taken together with our data, these observations underline the links between proteasome targets, drug resistance, quiescence entry and tumor dormancy in cancer patients. In contrast, the YAPdc-S127A mutant was not degraded by 5FU treatment (Figure 5B), because this mutation prevented the corresponding YAP phosphorylation and degradation through the phosphodegron. Thus, this S127A YAP mutant prevents both YAP degradation and 14-3-3 -mediated YAP cytoplasmic retention [7]. We reported here that YAPdc-S127A reversed 5FU-induced 5F31 quiescence through CREB phosphorylation and activation. In this context, CREB was phosphorylated at Ser133 after YAPdc ectopic transfection and 5FU treatment. This transcription factor is activated by several growth factors and cellular stress signals, such as 5FU chemotherapy. Indeed, CREB is the recognized substrate of several cellular kinases including PKA, PP90^RSK^, PKC, AKT, MSK1 and p38 stress-activated kinase [42]. Strikingly, we observed that CREB Ser133 phosphorylation was greatly enhanced in 5FU-treated YAPdc cells, showing that enhanced nuclear YAP potentiates CREB activation under 5FU exposure. Phosphorylation of CREB at Ser133 is a critical step in CREB activation as it promotes the recruitment of the transcriptional co-activator CBP and its paralog p300 [42]. Consistently, inhibition of the CREB/CBP complex by KG-501 in YAPdc cells restored both the entry into quiescence and Cyclin E1 down-regulation induced by 5FU, showing that oncogenic CREB played a pivotal role in YAPdc-mediated blockade of quiescence induced by 5FU. This observation agrees with the fact that CREB signaling promotes cell proliferation, notably by transcriptional activation of the cell cycle regulators Cyclins D1, A, B1 and most interestingly, Cyclin E1 [42, 43]. Molecular and signaling interactions between YAP and CREB were recently reported to generate mutual impacts in both stability and transcriptional activity of the two proteins [44]. Such a crosstalk is integrated in a signalosome comprising p38, CREB-P, the E3-ubiquitin ligase β-TrCP and the physical interactions between YAP and CREB. Since the YAPdc mutant escapes from proteasome degradation (*vide supra*), the nuclear abundance of ectopic YAP-S127A supports CREB activation and high Cyclin E1 levels under 5FU-treatment. In order to improve our understanding of the effective connectivity between YAP and CREB in colon cancer progression, we performed the bioinformatics evaluation of the genetic interactions between *YAP* and *CREB*, according to Amelio et al. [45]. Originally, the SynTarget web tool was designed to analyze the synergetic effect of two genes on survival outcome in patients with triple-negative breast cancer. In the present study, bioinformatics evaluation of 566 primary tumors resected from colon cancer patients (GSE 39582 dataset gathering transcriptomic analyses) revealed that the cumulative effects of High-YAP and High-CREB transcript levels correlated with a shorter overall survival (*p* = 0.00225). Interestingly, High-TAZ combined with High-CREB is associated with a detrimental impact on the survival of colon cancer patients (*p* = 0.00141), suggesting that TAZ is connected with CREB signaling pathways. As expected, a synergetic effect between *YAP* and *WWTR1* gene (encoding TAZ protein) expression on survival outcome was effective (*p* = 0.0032). In contrast, the *YAP* and *CCNE1* genes pair displayed no cumulative effect according SynTarget. In summary, our study indicates that potential synergies between YAP/TAZ and CREB are effective in primary colorectal tumors and liver metastases. So, we can assume that these transcriptional and post-transcriptional programs connected with signaling crosstalks participate in the multistep processes of the neoplastic conversion, drug resistance patterns and cancer patient survival.

Recent advances in the field indicate that the transcriptional coactivator YAP is functionally targeted by stress signals, oncogenic GPCR and Wnt signaling driving colon cancer progression [46, 47]. Thus, nuclear oncogenic YAP mediates tumor growth through potentiation of CREB signaling pathways induced by 5FU stress and proliferation/survival signals promoting YAP-dependent transcription of oncogenic pathways. In this scenario, one can postulate that YAP co-activator and its antagonistic Hippo tumor suppressive pathways are acting as molecular commutators at the proliferation/quiescence balance. This mechanism is likely to be involved in stemness, 5FU chemoresistance, dormancy and tumor recurrence. It is now accepted that 5FU- and oxaliplatin-resistant cancer cells display phenotypic changes consistent with stemness, EMT and invasion [1, 22, 48, 49]. Most interestingly, silencing of YAP in 5F31 cells led to a marked down-regulation of stemness markers (ALDH1A3, CD133 and LGR5) and sphere-forming capacity, suggesting that YAP is required for the maintenance of stem cell traits in 5FU-chemoresistant 5F31 cells. Our study on human liver metastases resected from colon cancer patients revealed that the YAP/TAZ level was highly correlated with the proliferation marker Ki-67, showing the crucial role of these two co-activators in growth of human colon liver metastases. In addition, YAP/TAZ was highly correlated with CTGF, supporting the notion that this growth factor is a critical component in the outgrowth of liver metastases. YAP-mediated CTGF expression was shown to be mainly involved in tumor stromal cell activation and stroma remodeling in human skin basal carcinoma [50]. The pleiotropic roles of the tumor stroma in cancer promotion, progression and metastasis are now well established. Recent studies highlight the reciprocal interplay between the tumor microenvironment and cancer cells in the determination of clinical outcomes and multiple forms of tumor chemoresistances [51]. Remarkably, we found that the combined YAP/TAZ transcript levels in liver metastases were highly correlated with reduced disease-free and overall survival, supporting the potential implication of YAP/TAZ signaling pathways in tumor relapse. Interestingly, in the subtype classification of colorectal cancers determined by Sadanandam and coworkers according to transcriptome analysis [52], *WWTR1, Cyr61* and *CTGF* genes were associated with the stem cell-like signature and subtype.

Finally, our studies support a key role for YAP/TAZ co-activators in growth and recurrence of colon cancer and argue that these co-activators and their molecular partners can be potent druggable targets to prevent tumor relapse. We expect that targeting YAP/TAZ and CREB signaling pathways and molecular interactions will help to identify new combination treatments for tumor dormancy prevention and elimination in colon cancer patients.

## MATERIALS AND METHODS

### Human colorectal cancer cell lines

HT29, RKO and HCT116 cell lines were purchased from the American Type Culture Collection. Chemoresistant clonal subpopulations 5F31 were isolated as previously described [53]. All cell lines were authenticated through the Short Tandem Repeat (STR) DNA profile analysis according to the procedures recommended by the ATCC Institute. All cell lines were cultivated in DMEM media containing 10% of heat-inactivated FBS, L-glutamine and penicillin/streptomycin.

### Pharmacological agents

5-Fluorouracil was obtained from the Hospital Pharmacy of Lille and treatment of the different cell lines was done accordingly to previous work of the laboratory [1]. KG-501 and Verteporfin (VP) were purchased from Sigma Aldrich and cells were treated for 48 hours at micro molar concentrations. For VP treatment, cells were treaded in the dark to avoid any issue due to photosensitivity of this molecule. Cellular toxicity (CT) of VP (5 and 10 μM) was < 5% in HCT116 cells and 7% in 5F31 cells, respectively. Clonogenic assays were performed in 6 wells-plate with low density cell seeding (5,000 cells per well). After two weeks of growing, cells were fixed with 4% paraformaldehyde and stained with a 0.4% violet crystal solution. The number of colonies was counted and the experiment is represented as a percentage of Colony Forming Unit (number of colonies/number of cells seeded per well).

### Patient tissue samples

70 liver metastases of colon adenocarcinoma and 30 healthy adjacent fragments were collected after surgery and stored in the Tumor Bank and Tissue Collection of the Pathology Department of Lille Hospital. Informed consent was obtained from all patients. Patient's characteristics are listed in Supplementary Table S1. The median follow up was 58.5 months (range, 31.7– 94.7 months). The median survival was 51.5 months (range, 28.7–69 months) and the disease free survival was 25.5 months (range, 10– 50 months). The 2-year postsurgical overall survival was 81.4% and the 2-year disease free survival was 50%. Half of this cohort (*n* = 35) received neoadjuvant 5-FU-based chemotherapy (FOLFOX, FOLFIRI, FOLFIRI with bevacizumab, XELODA, XELOX or XELOX plus bevacizumab).

### Transfection procedures

For transient inhibition of genes, we used Dharmacon smartpool siRNAs (with a working concentration of 15 or 30 nM of siRNA, listing of siRNAs in Supplementary Materials and Methods) and Dharmafect transfection media. Non-targeting siRNA control pools were used as negative controls.

### Expression of the constitutively active mutant YAPdc

5F31 cells were transfected with a plasmid encoding the dominant constitutive mutant Ser127Ala-YAP (Flag-YAPdc) or its corresponding empty vector as control (Addgene, reference 27370). Stable transfectants were selected with 1 μg/ml puromycin.

### Sphere formation

5F31 cells were plated at a density of 2,000 cells/ ml on plates coated with poly-2-hydroxyethylmetacrylate (0.5 mg/ml in ethanol) and cultured in serum-free DMEM/F12 medium supplemented with 20 ng/mL bFGF, 20 ng/ mL EGF and 1X B27. Spheres were counted after 7 days of culture.

### Western blotting and phospho-kinase array

Total extracts of cells were obtained with a RIPA-based buffer containing protease and phosphatase inhibitors (Roche). Subcellular fractionation was realized as previously described [1]. Western blot were carried out using 20 μg of protein lysates with the NuPage Electrophoresis and Iblot transfert systems (Life Technologies). The list of primary antibodies is described in the Supplementary Material and Methods and HRP-labelled secondary antibodies (Thermoscientific) were used accordingly to the primary antibody species. β-Actin was used as loading control for total extracts, SP-1 was used for labeling nuclear fractions. Western blot pictures were taken with the LAS 4000 camera (Fujifilm) and quantification were done with ImageJ software. Phosphokinase array (ARY003b) was done accordingly to manufacturer's protocol with 0.4 mg of protein lysate per membrane. The phospho-kinase array coordinates are: A1, A2: positive controls. A3, A4: p38α; A5, A6: ERK1/2; A7, A8: JNK pan; A9, A10: GSK-3α/β A13, A14:p53 (S392); A17, A18: positive control; B3, B4: EGFR (Y1086) ; B5, B6: MSK1/2; B7, B8: AMPKα1; B9, B10: Akt (S473); B11, B12: Akt (T308); B13, B14: p53 (S46); C1, C2: TOR; C3, C4: CREB; C5, C6: HSP27; C7, C8: AMPKα2; C9, C10: β-catenin; C11, C12: p70 S6 kinase; C13, C14: p53 (S15); C15, C16: c Jun (S63); D1, D2: Src; D3, D4: Lyn; D5, D6: Lck; D7, D8: STAT2; D9, D10: STAT5a; D11, D12: p70 S6 kinase (T421/S424); D13, D14: RSK1/2/3 D15, D16: eNOS (S1157); E1, E2: Fyn; E3, E4: Yes; E5, E6: Fgr; E7, E8: STAT6; E9, E10: STAT5b; E11, E12: STAT3; E13, E14: p27 (T198); E15, E16: PLC-γ1; F1, F2: Hck; F3, F4: Chk-2; F5, F6: FAK; F7, F8: PDGF Rβ; F9, F10: STAT5a/b; F11, F12: STAT3; F13, F14: WNK1; F15, F16: PYK2; F17, F18: negative controls; G1, G2: positive controls; G5, G6: negative controls.

### TEAD transcriptional activity

Cells at 50% confluence were transfected with the TEAD luciferase reporter plasmid 8XGTIIC-Luciferase (Addgene reference 34615) *vs* a control luciferase plasmid. After 48 hours post transfection, cells are lysed in Reporter Lysis Buffer (Promega) and luciferase activity was measured on the Mithras LB940 plate reader and normalized to protein concentration.

### Immunohistochemistry

Formalin-Fixed Paraffin embedded sections of 4 μm were deparaffinized through a series of xylen ethanol baths. Antigen retrieval was performed through microwaving in a 0.01 M citrate buffer for 30 minutes. After inactivation of endogenous peroxidases, primary antibodies were applied on the section overnight at 4°C. After the staining with primary and secondary peroxidase-labeled antibodies, the immunoreaction was visualized by the Ultraview-DAB system and slides were counterstained with hematoxylin. Specificity was checked by control staining performed in the absence of primary antibody and with positive tissue. Antibodies used are anti-YAP/TAZ antibody (Cell signaling 8418, dilution 1/200) or anti Ki-67 MIB1 (Dako M7240 dilution 1/50). Scoring and percentage of immunoreactive cells for each marker was done independently by two experienced pathologists from our team (F.R and E.L).

### Confocal microscopy

Cells were cultivated on Labtek chamber slides and treated with either siRNA or pharmacological drugs. After treatment, cells were fixed with 4% paraformaldehyde and permeabilized with a solution of Triton X100 diluted in PBS. PBS-BSA solution was used as blocking buffer and Ki-67, Cyclin E1 or YAP staining was realized by overnight incubation with primary antibodies (Santa Cruz Biotechnologies). After wash-up of primary antibodies, secondary antibodies labelled with Alexa Fluor dyes were used (Alexa Fluor 594, Life Technologies) and nuclei were labelled with Vectashield mounting media with DAPI (Vector). Pictures were taken on the BiCell platform of Lille on a Zeiss LSM 710 microscope at x40 or x63 magnification.

### mRNA expression

mRNAs were extracted from cultured cells or human tumors with the NucleoSpin kit (Macherey-Nagel). Retro-transcription was done on 1 μg of mRNA accordingly to the Advantage RT-for-PCR Kit protocol (Clontech). PCR was performed using SsoFastTM Evagreen Supermix kit following the manufacturer's protocol using the CFX96 real time PCR system (Bio-Rad). Primers used for this study are described within the Supplementary Material and Methods. To monitor any change in mRNA expression, we used the ΔΔCt method between one condition and a control condition after normalization with the housekeeper gene *RPLP0*. Each sample was done in triplicate. The expression level of YAP, TAZ, Cyr 61, CTGF, Ki-67 was assessed for all the 70 patients. The analysis of YAP and TAZ expression level led us to split our cohort into two groups, i.e. YAP-TAZ low (*n* = 42) and YAP-TAZ high (*n* = 28), according to the expression level of the co-activators within the metastatic tissue compared to adjacent non tumoral liver tissue. A patient with an expression level of YAP, TAZ or both transcripts over the threshold of 2 was enlisted in the YAP-TAZ high group.

### Flow cytometry

Cell viability and toxicity was analysed by exclusion of Propidium Iodide and flow cytometry. For cell cycle analysis, cell suspensions (200,000 cells per condition) were fixed with 70% ethanol at −20°C and incubated with 0.4 μg of anti-human Alexa 488-conjugated Ki67 antibody or matching 488-conjugated isotype (BD Biosciences) for 30 minutes on ice and for 30 minutes with 50 μg/ml propidium iodide (Sigma Aldrich) and 5 μg/ml RNase (Ambion) at room temperature. Reaction was stopped on ice and cells were analyzed on the Cyan ADP analyzer (Beckman Coulter). Cytometry data were analyzed with Kaluza software (Beckman Coulter).

### Syntarget Software

We analyzed the putative synergy between our molecular markers through the SynTarget Software (http://www.chemoprofiling.org/cgi-bin/GEO/cancertarget/web_run_CT.V0.S1.pl). We used the GSE 39582 dataset gathering transcriptomic analyses from 566 primary colon cancers.

### Statistical analyses

*In vitro* results were expressed as mean +/− Standard Deviation. The comparisons of groups were carried out using Mann–Whitney *U-test* or ANOVA. The relationships between clinico–morphological variables and YAP-TAZ in human samples were analyzed by the Fisher's or *X* ^2^-tests.

For univariate survival analysis we used the Kaplan–Meier method, the survival curves being compared by the log-rank test. For multivariate survival analysis, we used the Cox method. Variables related to postsurgical survival with a *p-value* of 0.05 in univariate analysis were included in Cox models.

For all the statistical tests and methods, a *P-value* of 0.05 was used for defining statistical significance and represented with *. When the *P value* was below 0.01 and 0.001, we used respectively the symbols ** and ***. Statistical analyses were carried out with GraphPad Prism v4 software (San Diego, CA, USA) and SPSS v23.0 software (Chicago, Ill, USA).

## SUPPLEMENTARY MATERIALS FIGURES AND TABLES



## References

[1] Touil Y, Igoudjil W, Corvaisier M, Dessein AF, Vandomme J, Monte D, Stechly L, Skrypek N, Langlois C, Grard G, Millet G, Leteurtre E, Dumont P (2014). Colon cancer cells escape 5FU chemotherapy-induced cell death by entering stemness and quiescence associated with the c-Yes/YAP axis. Clin Cancer Res.

[2] Vassilev A, Kaneko KJ, Shu H, Zhao Y, DePamphilis ML (2001). TEAD/TEF transcription factors utilize the activation domain of YAP65, a Src/Yes-associated protein localized in the cytoplasm. Genes Dev.

[3] Zhao B, Ye X, Yu J, Li L, Li W, Li S, Yu J, Lin JD, Wang CY, Chinnaiyan AM, Lai ZC, Guan KL (2008). TEAD mediates YAP-dependent gene induction and growth control. Genes Dev.

[4] Lamar JM, Stern P, Liu H, Schindler JW, Jiang ZG, Hynes RO (2012). The Hippo pathway target, YAP, promotes metastasis through its TEAD-interaction domain. Proc Natl Acad Sci U S A.

[5] Lei QY, Zhang H, Zhao B, Zha ZY, Bai F, Pei XH, Zhao S, Xiong Y, Guan KL (2008). TAZ promotes cell proliferation and epithelial-mesenchymal transition and is inhibited by the hippo pathway. Mol Cell Biol.

[6] Zhang K, Qi HX, Hu ZM, Chang YN, Shi ZM, Han XH, Han YW, Zhang RX, Zhang Z, Chen T, Hong W (2015). YAP and TAZ Take Center Stage in Cancer. Biochemistry.

[7] Zhao B, Li L, Tumaneng K, Wang CY, Guan KL (2010). A coordinated phosphorylation by Lats and CK1 regulates YAP stability through SCF(beta-TRCP). Genes Dev.

[8] Zhao B, Wei X, Li W, Udan RS, Yang Q, Kim J, Xie J, Ikenoue T, Yu J, Li L, Zheng P, Ye K, Chinnaiyan A (2007). Inactivation of YAP oncoprotein by the Hippo pathway is involved in cell contact inhibition and tissue growth control. Genes Dev.

[9] Kim NG, Koh E, Chen X, Gumbiner BM (2011). E-cadherin mediates contact inhibition of proliferation through Hippo signaling-pathway components. Proc Natl Acad Sci U S A.

[10] Wada K, Itoga K, Okano T, Yonemura S, Sasaki H (2011). Hippo pathway regulation by cell morphology and stress fibers. Development.

[11] Zhao D, Zhi X, Zhou Z, Chen C (2012). TAZ antagonizes the WWP1-mediated KLF5 degradation and promotes breast cell proliferation and tumorigenesis. Carcinogenesis.

[12] Overholtzer M, Zhang J, Smolen GA, Muir B, Li W, Sgroi DC, Deng CX, Brugge JS, Haber DA (2006). Transforming properties of YAP, a candidate oncogene on the chromosome 11q22 amplicon. Proc Natl Acad Sci U S A.

[13] Steinhardt AA, Gayyed MF, Klein AP, Dong J, Maitra A, Pan D, Montgomery EA, Anders RA (2008). Expression of Yes-associated protein in common solid tumors. Hum Pathol.

[14] Zhou D, Zhang Y, Wu H, Barry E, Yin Y, Lawrence E, Dawson D, Willis JE, Markowitz SD, Camargo FD, Avruch J (2011). Mst1 and Mst2 protein kinases restrain intestinal stem cell proliferation and colonic tumorigenesis by inhibition of Yes-associated protein (Yap) overabundance. Proc Natl Acad Sci U S A.

[15] Camargo FD, Gokhale S, Johnnidis JB, Fu D, Bell GW, Jaenisch R, Brummelkamp TR (2007). YAP1 increases organ size and expands undifferentiated progenitor cells. Curr Biol.

[16] Chan SW, Lim CJ, Guo K, Ng CP, Lee I, Hunziker W, Zeng Q, Hong W (2008). A role for TAZ in migration, invasion, and tumorigenesis of breast cancer cells. Cancer Res.

[17] Lian I, Kim J, Okazawa H, Zhao J, Zhao B, Yu J, Chinnaiyan A, Israel MA, Goldstein LS, Abujarour R, Ding S, Guan KL (2010). The role of YAP transcription coactivator in regulating stem cell self-renewal and differentiation. Genes Dev.

[18] Shao DD, Xue W, Krall EB, Bhutkar A, Piccioni F, Wang X, Schinzel AC, Sood S, Rosenbluh J, Kim JW, Zwang Y, Roberts TM, Root DE (2014). KRAS and YAP1 converge to regulate EMT and tumor survival. Cell.

[19] Hong JH, Hwang ES, McManus MT, Amsterdam A, Tian Y, Kalmukova R, Mueller E, Benjamin T, Spiegelman BM, Sharp PA, Hopkins N, Yaffe MB (2005). TAZ, a transcriptional modulator of mesenchymal stem cell differentiation. Science.

[20] Zhang H, Liu CY, Zha ZY, Zhao B, Yao J, Zhao S, Xiong Y, Lei QY, Guan KL (2009). TEAD transcription factors mediate the function of TAZ in cell growth and epithelial-mesenchymal transition. J Biol Chem.

[21] Fischer KR, Durrans A, Lee S, Sheng J, Li F, Wong ST, Choi H, El Rayes T, Ryu S, Troeger J, Schwabe RF, Vahdat LT, Altorki NK (2015). Epithelial-to-mesenchymal transition is not required for lung metastasis but contributes to chemoresistance. Nature.

[22] Sabbah M, Emami S, Redeuilh G, Julien S, Prevost G, Zimber A, Ouelaa R, Bracke M, De Wever O, Gespach C (2008). Molecular signature and therapeutic perspective of the epithelial-to-mesenchymal transitions in epithelial cancers. Drug Resist Updat.

[23] Cordenonsi M, Zanconato F, Azzolin L, Forcato M, Rosato A, Frasson C, Inui M, Montagner M, Parenti AR, Poletti A, Daidone MG, Dupont S, Basso G (2011). The Hippo transducer TAZ confers cancer stem cell-related traits on breast cancer cells. Cell.

[24] Lin CH, Pelissier FA, Zhang H, Lakins J, Weaver VM, Park C, LaBarge MA (2015). Microenvironment rigidity modulates responses to the HER2 receptor tyrosine kinase inhibitor lapatinib via YAP and TAZ transcription factors. Mol Biol Cell.

[25] Lin L, Sabnis AJ, Chan E, Olivas V, Cade L, Pazarentzos E, Asthana S, Neel D, Yan JJ, Lu X, Pham L, Wang MM, Karachaliou N (2015). The Hippo effector YAP promotes resistance to RAF- and MEK-targeted cancer therapies. Nat Genet.

[26] Liu-Chittenden Y, Huang B, Shim JS, Chen Q, Lee SJ, Anders RA, Liu JO, Pan D (2012). Genetic and pharmacological disruption of the TEAD-YAP complex suppresses the oncogenic activity of YAP. Genes Dev.

[27] Clevers H (2011). The cancer stem cell: premises, promises and challenges. Nat Med.

[28] Wells A, Griffith L, Wells JZ, Taylor DP (2013). The dormancy dilemma: quiescence versus balanced proliferation. Cancer Res.

[29] Cioffi M, Trabulo SM, Sanchez-Ripoll Y, Miranda-Lorenzo I, Lonardo E, Dorado J, Reis Vieira C, Ramirez JC, Hidalgo M, Aicher A, Hahn S, Sainz B, Heeschen C (2015). The miR-17-92 cluster counteracts quiescence and chemoresistance in a distinct subpopulation of pancreatic cancer stem cells. Gut.

[30] Holohan C, Van Schaeybroeck S, Longley DB, Johnston PG (2013). Cancer drug resistance: an evolving paradigm. Nat Rev Cancer.

[31] Zanconato F, Forcato M, Battilana G, Azzolin L, Quaranta E, Bodega B, Rosato A, Bicciato S, Cordenonsi M, Piccolo S (2015). Genome-wide association between YAP/TAZ/TEAD and AP-1 at enhancers drives oncogenic growth. Nat Cell Biol.

[32] Hua K, Yang W, Song H, Song J, Wei C, Li D, Fang L (2015). Up-regulation of miR-506 inhibits cell growth and disrupt the cell cycle by targeting YAP in breast cancer cells. Int J Clin Exp Med.

[33] Caldon CE, Musgrove EA (2010). Distinct and redundant functions of cyclin E1 and cyclin E2 in development and cancer. Cell Div.

[34] Hinds PW, Mittnacht S, Dulic V, Arnold A, Reed SI, Weinberg RA (1992). Regulation of retinoblastoma protein functions by ectopic expression of human cyclins. Cell.

[35] Geng Y, Yu Q, Sicinska E, Das M, Schneider JE, Bhattacharya S, Rideout WM, Bronson RT, Gardner H, Sicinski P (2003). Cyclin E ablation in the mouse. Cell.

[36] Geng Y, Lee YM, Welcker M, Swanger J, Zagozdzon A, Winer JD, Roberts JM, Kaldis P, Clurman BE, Sicinski P (2007). Kinase-independent function of cyclin E. Mol Cell.

[37] Parisi T, Beck AR, Rougier N, McNeil T, Lucian L, Werb Z, Amati B (2003). Cyclins E1 and E2 are required for endoreplication in placental trophoblast giant cells. EMBO J.

[38] Campaner S, Viale A, De Fazio S, Doni M, De Franco F, D’Artista L, Sardella D, Pelicci PG, Amati B (2013). A non-redundant function of cyclin E1 in hematopoietic stem cells. Cell Cycle.

[39] Geng Y, Yu Q, Whoriskey W, Dick F, Tsai KY, Ford HL, Biswas DK, Pardee AB, Amati B, Jacks T, Richardson A, Dyson N, Sicinski P (2001). Expression of cyclins E1 and E2 during mouse development and in neoplasia. Proc Natl Acad Sci U S A.

[40] Takeishi S, Matsumoto A, Onoyama I, Naka K, Hirao A, Nakayama KI (2013). Ablation of Fbxw7 eliminates leukemia-initiating cells by preventing quiescence. Cancer Cell.

[41] Siu KT, Rosner MR, Minella AC (2012). An integrated view of cyclin E function and regulation. Cell Cycle.

[42] Mayr B, Montminy M (2001). Transcriptional regulation by the phosphorylation-dependent factor CREB. Nat Rev Mol Cell Biol.

[43] Impey S, McCorkle SR, Cha-Molstad H, Dwyer JM, Yochum GS, Boss JM, McWeeney S, Dunn JJ, Mandel G, Goodman RH (2004). Defining the CREB regulon: a genome-wide analysis of transcription factor regulatory regions. Cell.

[44] Wang J, Ma L, Weng W, Qiao Y, Zhang Y, He J, Wang H, Xiao W, Li L, Chu Q, Pan Q, Yu Y, Sun F (2013). Mutual interaction between YAP and CREB promotes tumorigenesis in liver cancer. Hepatology.

[45] Amelio I, Tsvetkov PO, Knight RA, Lisitsa A, Melino G, Antonov AV (2016). SynTarget: an online tool to test the synergetic effect of genes on survival outcome in cancer. Cell Death Differ.

[46] Guo X, Zhao B (2013). Integration of mechanical and chemical signals by YAP and TAZ transcription coactivators. Cell Biosci.

[47] Azzolin L, Panciera T, Soligo S, Enzo E, Bicciato S, Dupont S, Bresolin S, Frasson C, Basso G, Guzzardo V, Fassina A, Cordenonsi M, Piccolo S (2014). YAP/TAZ incorporation in the beta-catenin destruction complex orchestrates the Wnt response. Cell.

[48] Yang AD, Fan F, Camp ER, van Buren G, Liu W, Somcio R, Gray MJ, Cheng H, Hoff PM, Ellis LM (2006). Chronic oxaliplatin resistance induces epithelial-to-mesenchymal transition in colorectal cancer cell lines. Clin Cancer Res.

[49] Gespach C (2010). Stem cells and colon cancer: the questionable cancer stem cell hypothesis. Gastroenterol Clin Biol.

[50] Quan T, Xu Y, Qin Z, Robichaud P, Betcher S, Calderone K, He T, Johnson TM, Voorhees JJ, Fisher GJ (2014). Elevated YAP and its downstream targets CCN1 and CCN2 in basal cell carcinoma: impact on keratinocyte proliferation and stromal cell activation. Am J Pathol.

[51] Castells M, Thibault B, Delord JP, Couderc B (2012). Implication of tumor microenvironment in chemoresistance: tumor-associated stromal cells protect tumor cells from cell death. Int J Mol Sci.

[52] Sadanandam A, Lyssiotis CA, Homicsko K, Collisson EA, Gibb WJ, Wullschleger S, Ostos LC, Lannon WA, Grotzinger C, Del Rio M, Lhermitte B, Olshen AB, Wiedenmann B (2013). A colorectal cancer classification system that associates cellular phenotype and responses to therapy. Nat Med.

[53] Lesuffleur T, Kornowski A, Luccioni C, Muleris M, Barbat A, Beaumatin J, Dussaulx E, Dutrillaux B, Zweibaum A (1991). Adaptation to 5-fluorouracil of the heterogeneous human colon tumor cell line HT-29 results in the selection of cells committed to differentiation. Int J Cancer.

