# Supplementary material for: Regulation of cellular quiescence by YAP/TAZ and Cyclin E1 in colon cancer cells: Implication in chemoresistance and cancer relapse
Source: Oncotarget. 2016 Aug 4;7(35):56699–712. doi: 10.18632/oncotarget.11057 (PMC5302946; doi:10.18632/oncotarget.11057)
Supplement: Supplementary file 1 [file oncotarget-07-56699-s001.pdf]

Regulation of cellular quiescence by YAP/TAZ and Cyclin E1 in colon cancer cells: Implication in chemoresistance and cancer relapse

Supplementary Materials

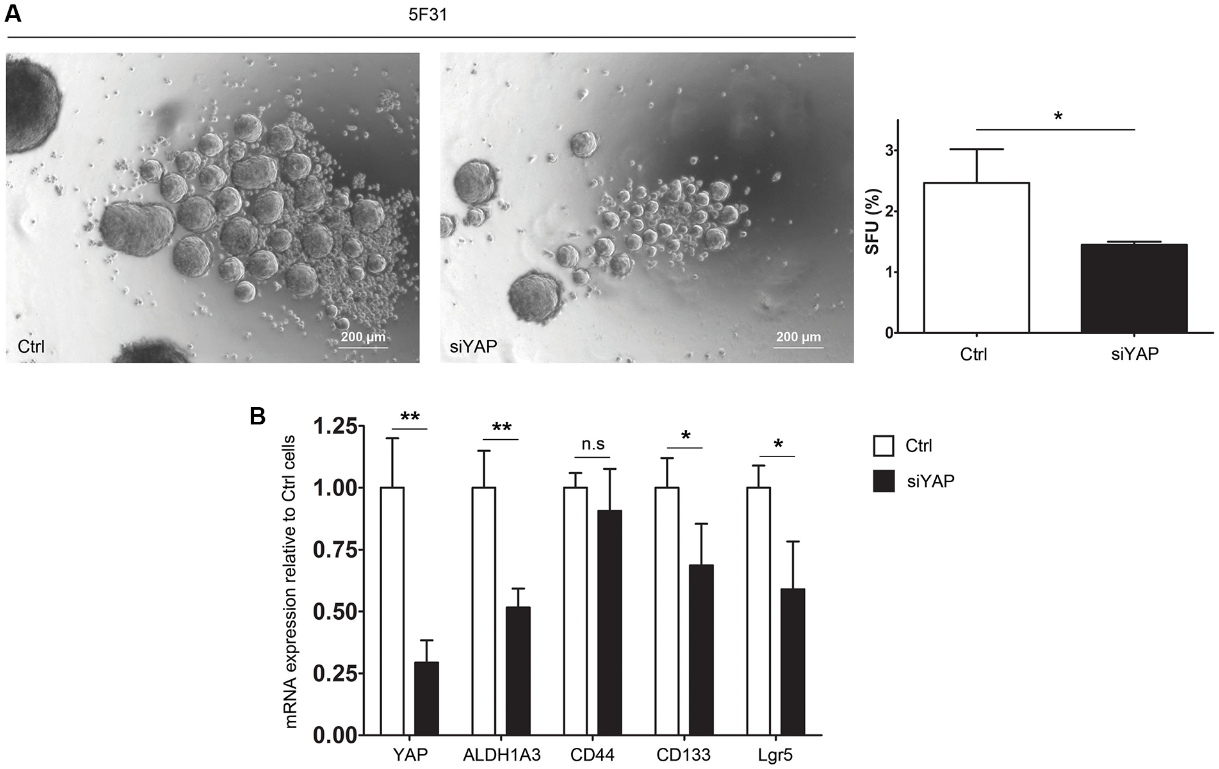

Supplementary Figure S1: YAP knock-down alleviates stemness traits in 5F31 cells. Sphere Forming Unit (SFU) assay of Control (Ctrl) and YAP-silenced 5F31 cells performed one week after transfection. SFU is the ratio of the number of spheres formed *per* the number of cells seeded per well. (**A**) Pictures were taken at 20× magnification. (**B**) Expression of the transcripts encoding YAP, ALDH1A3, CD44, CD133 and Lgr5 after YAP silencing.

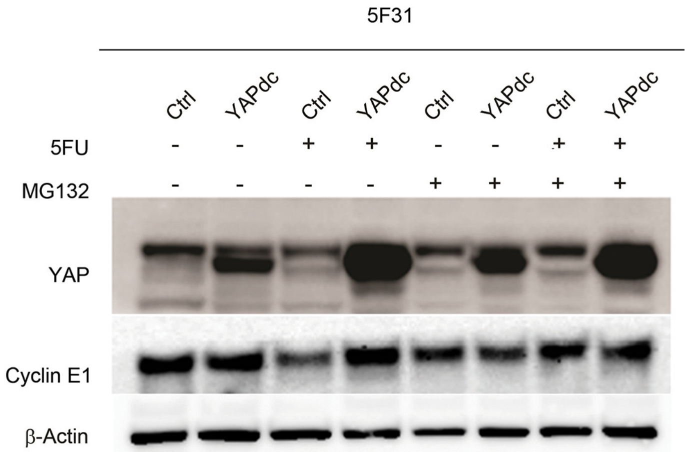

Supplementary Figure S2: Proteasome-dependent degradation of YAP and Cyclin E1 in 5F31 cells after 5FU-treatment. Western blot analysis of YAP and Cyclin E1 in control (Ctrl) and YAPdc-transfected 5F31 cells cultured for 96 hours in the presence or absence of 5FU and/or pretreatment with MG132 (1 μM for 8 hours).

Supplementary Table S1: Clinical and pathological features of metastatic liver samples correlated to YAP-TAZ expression

	YAP-TAZ low	YAP-TAZ high	*p*
Number of patients	42	28	
Age at surgery			
< 60 years	21 (50%)	8 (28.6%)	0.075
> 60 years	21 (50%)	20 (71.4%)	
Primary tumor site			
Right colon	8 (19%)	8 (28.6%)	0.249
Left colon	23 (54.8%)	17 (60.7%)	
Rectum	11 (26.2%)	3 (10.7%)	
Synchronous metastasis			
Yes	13 (31%)	13 (48.1%)	0.150
No	29 (69%)	15 (51.9%)	
Bilobar metastasis			
Yes	2 (4.8%)	1 (3.6%)	0.810
No	40 (95.2%)	27 (96.4%)	
T			
Tis. T1. T2	7 (21.2%)	3 (13.6%)	0.475
T3. T4	26 (78.8%)	19 (86.4%)	
Tx	9 (11.9%)	6 (3.5%)	
N			
N0	19 (45.2%)	10 (35.7%)	0.678
N+	14 (33.3%)	12 (42.9%)	
Nx	9 (21.4%)	6 (21.4%)	
Neo adjuvant chemotherapy	17 (40.5%)	17 (60.7%)	0.097
FOLFOX	11	9	0.589
FOLFIRI	6	8	0.143
XELODA	2	0	0.241
With bevacizumab	6 (13.3%)	9 (28.6%)	0.074
Without bevacizumab	36 (85.7%)	19 (71.4%)	

*Values are median (interquartile range).

Supplementary Table S2: Univariate disease free survival analysis

	Number of patients	DFS (months)*	Log-rank p value
YAP-TAZ			
High	28 (40%)	15 [8–31]	**0.008**
Low	42 (60%)	30.5 [14–60]	
Age at surgery			
< 60 (Years)	29 (41.4%)	28 [9–45]	0.832
> 60 (Years)	41 (58.6%)	23 [13–49]	
Primary tumor site			
Right colon	16 (22.9%)	29 [14.5–53]	0.168
Left colon	40 (57.1%)	16.5 [8–35]	
Rectum	14 (20%)	37.5 [20–60]	
Synchronous metastasis			
Yes	26 (37.1%)	15 [8–45]	**0.024**
No	44 (62.9%)	29 [15–60]	
Bilobar metastasis			
Yes	3 (4.3%)	23 [9–45]	0.762
No	67 (95.7%)	24 [10–53]	
T			
Tis. T1. T2	10 (18.1%)	19 [14–49]	0.901
T3. T4	45 (81.9%)	24 [9–45]	
N			
N0	29 (52.7%)	32 [15–59]	0.098
N+	26 (47.3%)	15 [8–31]	
Neo adjuvant chemotherapy			
Yes	34 (48.6%)	15 [8–35]	**0.040**
No	36 (51.4%)	30 [15–60]	

*Values are median [interquartile range].

Supplementary Table S3: Multivariate disease free survival analysis

	p value	Hazard ratio	95% confidence intervalle
**YAP-TAZ**	**0.045**	**1.979**	**1.014 to 3.860**
Synchronous metastasis	0.115	1.690	0.880 to 3.248
Neoadjuvant chemotherapy	0.184	1.570	0.807 to 3.054

Introduction when p univariate analysis, < 0.05.

Supplementary Table S4 : Univariate overall survival analysis

	Number of patients	OS (months)*	Log-rank *p* value
YAP-TAZ			
High	28 (40%)	31 [21–61]	**0.040**
Low	42 (60%)	58 [32–73]	
Age at surgery			
< 60 (Years)	29 (41.4%)	54 [32–69]	0.580
> 60 (Years)	41 (58.6%)	44 [29–67]	
Primary tumor site			
Right colon	16 (22.9%)	44 [22–67]	0.409
Left colon	40 (57.1%)	36 [27–65]	
Rectum	14 (20%)	62 [44–77]	
Synchronous metastasis			
Yes	26 (37.1%)	54 [22–61]	0.419
No	44 (62.9%)	50 [31–69]	
Bilobar metastasis			
Yes	3 (4.3%)	77 [12–79]	0.514
No	67 (95.7%)	50 [29–68]	
T			
Tis. T1. T2	10 (18.1%)	57 [22–73]	0.571
T3. T4	45 (81.9%)	35 [29–67]	
N			
N0	29 (52.7%)	54 [43–69]	0.109
N+	26 (47.3%)	57 [30–73]	
Neo adjuvant chemotherapy			
Yes	34 (48.6%)	35 [21–69]	0.386
No	36 (51.4%)	53 [31–69]	

*Values are median [interquartile range].

Supplementary Table S5: Cox model regression for overall survival analysis

	p value	Hazard ratio	95% confidence intervalle
YAP-TAZ	**0.045**	**2.058**	**1.018 to 4.161**

introduction when p univariate analysis, < 0.05.
